# Antidepressant effect of repetitive transcranial magnetic stimulation is not impaired by intake of lithium or antiepileptic drugs

**DOI:** 10.1007/s00406-021-01287-3

**Published:** 2021-07-03

**Authors:** T. Hebel, M. A. Abdelnaim, M. Deppe, P. M. Kreuzer, A. Mohonko, T. B. Poeppl, R. Rupprecht, B. Langguth, M. Schecklmann

**Affiliations:** 1 Department of Psychiatry and Psychotherapy, University Regensburg, Universitätsstraße 84, 93053 Regensburg, Germany; 2 Psychiatric University Hospital Zürich, Zürich, Switzerland; 3 Department of Psychiatry, Psychotherapy, and Psychosomatics, Medical Faculty, RWTH Aachen University, Aachen, Germany

**Keywords:** Depression, Repetitive transcranial magnetic stimulation, rTMS, Lithium, Lamotrigine, Valproic acid, Mood stabilizer

## Abstract

**Introduction:**

The effect of concomitant medication on repetitive transcranial magnetic stimulation (rTMS) outcomes in depression remains understudied. Recent analyses show attenuation of rTMS effects by antipsychotic medication and benzodiazepines, but data on the effects of antiepileptic drugs and lithium used as mood stabilizers or augmenting agents are sparse despite clinical relevance. Preclinical electrophysiological studies suggest relevant impact of the medication on treatment, but this might not translate into clinical practice. We aimed to investigate the role of lithium (Li), lamotrigine (LTG) and valproic acid (VPA) by analyzing rTMS treatment outcomes in depressed patients.

**Methods:**

299 patients with uni- and bipolar depression treated with rTMS were selected for analysis in respect to intake of lithium, lamotrigine and valproic acid. The majority (*n* = 251) were treated with high-frequency (10–20 Hz) rTMS of the lDLPFC for an average of 17 treatment sessions with a figure-of-8 coil with a MagVenture system aiming for 110% resting motor threshold, and smaller groups of patients were being treated with other protocols including intermittent theta-burst stimulation and bilateral prefrontal and medial prefrontal protocols. For group comparisons, we used analysis of variance with the between-subjects factor group or Chi-Square Test of Independence depending on the scales of measurement. For post-hoc tests, we used least significant difference (LSD). For differences in treatment effects between groups, we used an ANOVA with the between-subjects factor group (groups: no mood stabilizer, Li, LTG, VPA, Li + LTG) the within-subjects factor treatment (pre vs. post treatment with rTMS) and also Chi-Square Tests of independence for response and remission.

**Results:**

Overall, patients showed an amelioration of symptoms with no significant differences for the main effect of group and for the interaction effect treatment by group. Based on direct comparisons between the single groups taking mood stabilizers against the group taking no mood stabilizers, we see a superior effect of lamotrigine, valproic acid and combination of lithium and lamotrigine for the response and remission rates. Motor threshold was significantly and markedly higher for patients taking valproic acid.

**Conclusion:**

Being treated with lithium, lamotrigine and valproic acid had no relevant influence on rTMS treatment outcome. The results suggest there is no reason for clinicians to withhold or withdraw these types of medication from patients who are about to undergo a course of rTMS. Prospective controlled work on the subject is encouraged.

## Introduction

The necessity of adding innovative treatment strategies for the treatment of Major Depressive Disorder arises from the unsatisfactory state of purely pharmacological and psychotherapeutic treatment, which leaves a significant number of patients failing to reach remission [35].

Non-invasive brain stimulation by repetitive transcranial magnetic stimulation (rTMS) is an increasingly common, safe, and evidence-based adjunct therapy [20, 24, 28]. The most encouraging evidence for the use of rTMS in depression has come from large sham-controlled trials in which patients were required to be medication-free while receiving treatment [8, 26]. While methodologically sound from a scientific perspective, translating the results from such trials into everyday clinical practice can be challenging, as patients are rarely referred to rTMS services as a first-line treatment and are more often than not taking a number of psychiatric drugs [25].

There is growing evidence that concomitant use of medication can have influence on the clinical effectiveness of rTMS. For example, intake of benzodiazepines or antipsychotics lowers the efficacy [12, 15], although for the case of antipsychotics there is also evidence to the contrary [30]. For the case of the benzodiazepine lorazepam, retrospective analysis has found its [5] use to be associated with decreased effectiveness and response rates to rTMS. This finding is compatible with preclinical considerations, as the anti-depressive action of rTMS may depend crucially on the propagation of the stimulation effects from the primary rTMS target site to connected downstream neurocircuitries [5]. This propagation process in turn can be attenuated by benzodiazepines, such as midazolam [6], alprazolam or diazepam [27] in human subjects. However, pooled analysis by Fitzgerald et al. from two clinical trials could identify no difference in clinical depression outcomes between patients taking versus not taking benzodiazepines [7].

Other drugs commonly used in depressed patients have been studied less so far.

Lithium is one of the most studied and effective drugs in treating bipolar disorder, preventing both relapses of mania and depression and is counted among the “mood stabilizers” [23, 34]. Its use is not limited to bipolar disorder, and it is widely prescribed as an augmentation agent in unipolar depression with pronounced anti-suicidal effects [34].

Evidence from healthy volunteers suggests that lithium modulates corticospinal excitability and brain stimulation induced cortical plasticity [14, 36]. However, the consequences for brain stimulation practice remain elusive, especially considering that the effects of lithium “are most pronounced in the presence of pathology” [23], limiting the applicability of data from healthy volunteers.

Antiepileptic drugs are prescribed not only for epilepsy, but also for psychiatric disorders. Amongst the antiepileptic drugs, valproic acid (VPA), carbamazepine and lamotrigine (LTG) have been found to be of particular usefulness in the treatment of bipolar disorder [1]. However, clinicians have also begun testing the usefulness of these drugs in augmentation of unipolar depression [9–11, 32].

Regarding electrophysiological parameters, there is evidence from preclinical studies in healthy volunteers that LTG can elevate motor threshold, perhaps without changing intracortical excitability [19, 22, 37] and can modulate so called TMS-induced long-term potentiation-like plasticity [4, 13].

For VPA, studies in healthy volunteers found no effect on motor threshold [22, 38] or other TMS-derived electrophysiological parameters [38]. More studies have been performed on epileptic patients, but their applicability to the subject seems even more limited as the drug effects in epileptic patients probably “represent a correction of intrinsic defects of the epileptic brain” [33].

It must be stressed once again that the majority of these findings come from application of brief, research-protocol TMS interventions and single-dose drug challenges in healthy subjects and the derived electrophysiological parameters mostly refer to the motor system.

Therefore, there remains a substantial gap to bridge between these preclinical findings and the treatment of depressed patients receiving daily rTMS and daily medication over the course of weeks [15, 25]. We aimed to investigate the role of Li, LTG and VPA by analyzing rTMS treatment outcomes in depressed patients taking versus not taking these drugs in a retrospective naturalistic study. While such a study design cannot elucidate the molecular mechanisms of rTMS or the drugs in question, it can offer some insight into the important clinical question whether these drugs interfere with rTMS treatment in a clinically meaningful way.

## Methods

A cohort of patients with depression were treated with rTMS in the Center for Neuromodulation Regensburg (Germany) between 2002 and 2017. Patients gave written informed consent to treatment. The retrospective analysis of clinical data was approved by the local ethics committee of the University of Regensburg (16-104-0223) and is in accordance with the ethical standards laid down in the Declaration of Helsinki. We have reported on this sample before with regards to other psychotropic medication classes and their effects on rTMS outcomes [5, 12].

The inclusion criteria were: naïve to rTMS (only the patient’s first treatment with rTMS was considered), diagnosis of depression according to ICD-10 of F31-F33, a completed Hamilton Depression Rating Scale with 21 items (HDRS-21) at beginning and at the end of the rTMS treatment and absence of a serious somatic illness. Both in- and outpatients were included. Based on these criteria, a sample of 299 patients could be pre-selected for this analysis. Most of the patients did not take any mood stabilizer (*n* = 188), followed by a large group of patients taking lithium (*n* = 65) and smaller groups (*n* < 20) taking other mood stabilizers (one or two). No single patient took carbamazepine. Two patients taking lithium and valproate and two patients taking lamotrigine and valproate were excluded from analysis due to small and thus not representative sample size (Table 1).

**Table 1:** Characteristics of patients with depression taking vs. not taking mood stabilizers (data in brackets represent 95% confidence intervals; categorial variables are presented in absolute and relative frequencies)

	No mood stabilizer (*n* = 188)	Li (*n* = 65)	LTG (*n* = 18)	VPA (*n* = 11)	Li + LTG (*n* = 13)	Statistics for group contrasts
Age (years)	47.3 [45.5; 49.1]	47.8 [44.6; 51.1]	53.2 [47.2; 59.2]	48.3 [41.5; 55.1]	49.0 [42.6; 55.4]	*F* = 0.954; d*f* = 4290; *p* = 0.433
Sex (female/male)	99/89 (53/47[%])	30/35 (46/54[%])	11/7 ([61/39%])	7/4 (64/36[%])	11/2 (85/15[%])	*χ*^2^ = 7.397; d*f* = 4; *p* = 0.116
Resting motor threshold	43.0 [41.7; 44.4]	43.5 [41.2; 45.7]	42.4 [37.1; 47.8]	52.1 [44.1; 60.1]	41.7 [38.3; 45.1]	*T* = 2.608; d*f* = 4290; *p* = 0.036*
Stimulation intensity	45.0 [43.8; 46.1]	45.3 [43.2; 47.3]	43.6 [38.9; 48.2]	52.5 [47.4; 57.5]	44.8 [40.8; 48.7]	*F* = 2.392; d*f* = 4290; *p* = 0.051(*)
Number of pulses per session	1986 [1955; 2017]	2003 [1965; 2040]	2044 [1980; 2108]	1909 [1706; 2111]	2000 [n.a.; n.a.]	*F* = 0.891; d*f* = 4290; *p* = 0.469
Number of sessions per patient/treatment	17.2 [16.2; 18.1]	17.6 [15.8; 19.5]	17.6 [14.4; 20.8]	18.6 [15.2; 21.9]	15.5 [12.8; 18.3]	*F* = 0.812; d*f* = 4290; *p* = 0.812
HDRS-21 baseline	23.4 [22.4; 24.3]	23.7 [21.9; 25.5]	24.8 [22.2; 27.5]	25.0 [21.3; 28.8]	20.2 [16.8; 23.6]	*F* = 1.123; d*f* = 4290; *p* = 0.346
ICD-10 type of depression (F31/F32/F33)	3/68/117 (2/36/62[%])	9/12/44 (14/18/68[%])	5/1/12 (28/6/66[%])	4/2/4 (40/20/40[%])	1/3/8 (8/25/67[%])	*χ*^2^ = 46.748; d*f* = 8; *p* < 0.001*
ICD-10 severity of depression (mild + moderate/ severe/psychotic)	31/126/8 (19/76/5[%])	6/52/3 (10/85/5[%])	5/12/1 (28/67/5[%])	2/3/2 (29/42/29[%])	2/9/0 (18/82/0[%])	*χ*^2^ = 13.411; d*f* = 8; *p* = 0.098(*)
Response rate [yes/no] (relative frequency of responders)	55/133 (29/71[%])	17/48 (26/74[%])	7/11 (39/61[%])	4/7 (36/64[%])	5/8 (39/61[%])	*χ*^2^ = 1.842; d*f* = 4; *p* = 0.765
Remission rate (yes/no)	52/136 (28/72[%])	16/49 (25/75[%])	5/13 (28/72[%])	4/7 (36/64[%])	6/7 (46/54[%])	*χ*^2^ = 2.876; d*f* = 4; *p* = 0.579
Percentage change from pre to post treatment for HDRS-21	29.5 [24.7; 34.3]	27.0 [18.7; 35.3]	37.6 [19.1; 56.0]	39.3 [11.6; 67.0]	46.5 [31.7; 61.3]	*F* = 1.344; d*f* = 4,290; *p* = 0.254
Intake of antipsychotics (yes/no)	103/85 (55/45[%])	47/18 (72/28[%])	14/4 (78/22[%])	6/5 (55/45[%])	9/4 (69/31[%])	*χ*^2^ = 9.197; d*f* = 4; *p* = 0.056 (*)
Intake of benzodiazepines (yes/no)	54/134 (29/71[%])	33/32 (51/49[%])	10/8 (56/44[%])	2/9 (18/82[%])	4/9 (31/69[%])	*χ*^2^ = 15.189; d*f* = 4; *p* = 0.004

*Significant and (*) near significant differences between groups

Exact numbers and also descriptive sample characteristics of the remaining 295 patients can be seen in Table 1 (see results). Different study protocols were used—the majority of patients (*n* = 251) were treated with high-frequency protocols over the left DLPFC (227 with 20 Hz and 2000 pulses, 15 with 10 Hz and 2000 pulses, 8 with 10 Hz and 1000 pulses, 1 with 1 Hz and 2000 pulses). Four patients were stimulated over the right DLPFC (three with 20 Hz and 2000 pulses, 1 with 1 Hz and 1000 pulses), 12 over the medial prefrontal cortex with 10 Hz and 2000 pulses and 28 were stimulated over both the left and right DLPFC in consecutive order (12 with 1 Hz right followed by 10 Hz left, each site with 1000 pulses; 16 with cTBS right followed by iTBS left, each site with 1200 pulses). Average number of treatment days was 17 and treatment was performed 5 days a week with no treatment on weekends. High-frequency protocols were applied with 50 pulses in a train and an inter-train interval of 25 s. For low-frequency protocols, no pauses were included. Each treatment was performed with a MagVenture system (MagVenture Inc., USA) using a figure-of-8 coil (except a double cone coil for medial prefrontal cortex stimulation) aiming for a target treatment intensity of 110% resting motor threshold (except TBS protocols (80% resting motor threshold) and medial prefrontal cortex (100% resting motor threshold)). The upper limit of treatment intensity was set to 60% of stimulator output for safety and tolerability reasons.

All data were analyzed using SPSS (IBM Corp., USA; Version 24.0.0.0). The significance level was set at *p* < 0.05. For group comparisons, we used analysis of variance (ANOVA) with the between-subjects factor group (groups: no mood stabilizer, Li, LTG, VPA, Li + LTG) or Chi-Square-Test of Independence depending on the scales of measurement. For post hoc tests, we used least significant difference (LSD). For differences in treatment effects between groups, we used an ANOVA with the between-subjects factor group (see above) and the within-subjects factor treatment (pre vs. post treatment with rTMS) and also Chi-Square-Tests of independence for response (decrease of HDRS score of at least 50% from pre to post rTMS) and remission (HDRS score at end of treatment below 11 points) rates. Results of chi-square tests were replicated by repeating them with the Fisher’s exact test for reasons of low cell frequencies.

Based on these data, number needed to treat (NNT) and number needed to harm (NNH) were calculated (Lenhard W, 2016, J, 1988). To control for effects of taking mood stabilizers at all and to control for effects of age, gender, intake of benzodiazepines, intake of antipsychotics, and type of depression, we repeated the ANOVA once with the factor group (no mood stabilizer vs. mood stabilizer) and once with the covariates age, gender, intake of benzodiazepines (yes, no), intake of antipsychotics (yes, no), and type of depression.

Antidepressive treatment outcome was assessed by the Hamilton Depression Rating Scale with 21 items (HDRS-21).

## Results

Groups did not differ with respect to age, sex, number of treatment sessions, number of pulses per session and baseline depressive symptoms (HDRS), but differed with respect to resting motor threshold, stimulation intensity (near significant), type and severity (near significant) of depression.

Groups differed also (near) significantly with respect to the intake of benzodiazepines and antipsychotics (see Table 1) showing increased intake of antipsychotics in the group of patients taking Li, LTG and Li + LTG and also increased intake of benzodiazepines in the group of patients taking Li and LTG.

Post hoc LSD tests indicated significant increased resting motor threshold and stimulation intensity for the group of patients taking VPA in contrast to the other groups (all *p*-values < 0.05 (see Fig. 3). As seen in the scatter plot, there was one outlier (see Fig. 3a). Excluding this outlier from the ANOVA would result in a non-significant main effect of group (*F* = 1.413; d*f* = 4289; *p* = 0.230).

All other post hoc groups’ contrasts were not significant. Descriptive analyses of group differences for type of depression showed increased number of bipolar patients in the groups taking mood stabilizer and decreased number of patients with bipolar depression in the group taking no mood stabilizer. For severity of depression, there was no clear systematic unequal distribution for the single groups.

**Fig. 1:**
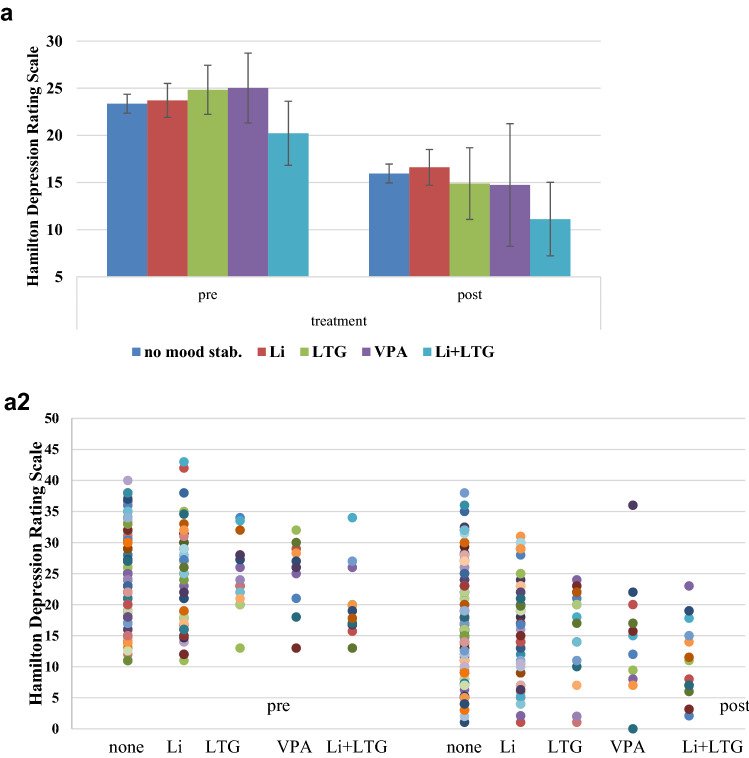

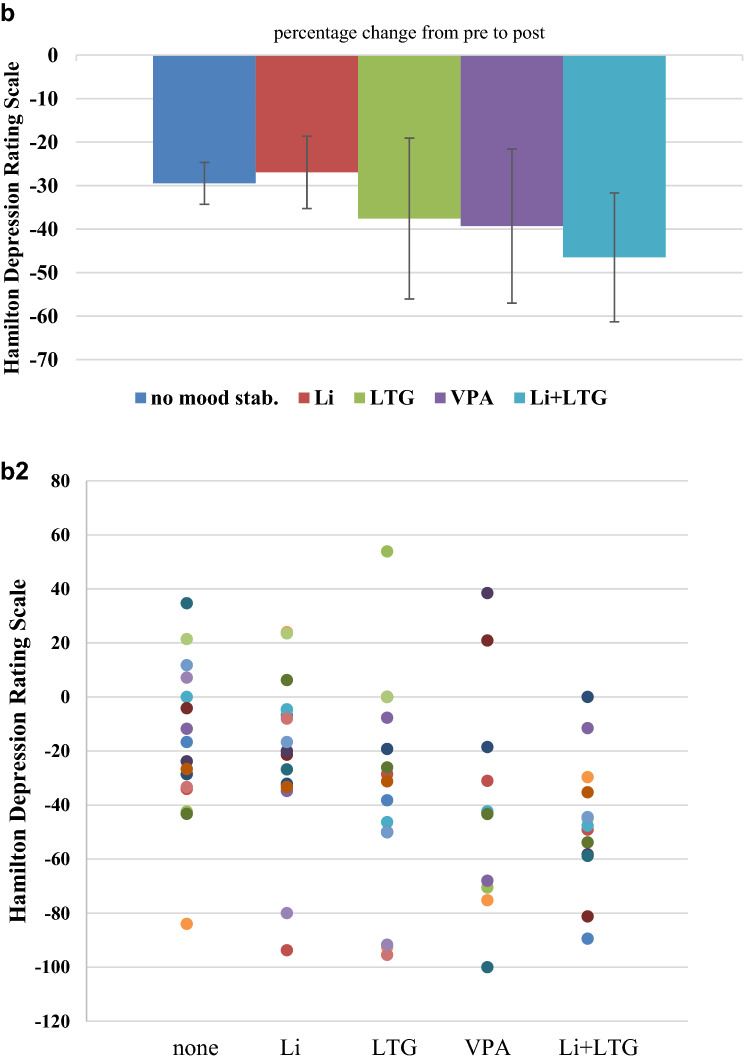
**a** Changes in HDRS-21 sum score from pre- to post-repetitive transcranial magnetic stimulation for patients taking different medication. Error bars represent 95% confidence intervals. **a2** Corresponding scatter plot. **b** Changes in HDRS scores expressed as percentual changes from pre to post treatment for patients taking different medication. Error bars represent 95% confidence intervals. **b2** Corresponding scatter plot

Overall, patients showed an amelioration of symptoms as indicated by a significant effect of treatment (*F* = 118.539; d*f* = 1290; *p* < 0.001) with no significant differences for the main effect of group (*F* = 1.583; d*f* = 4290; *p* = 0.179) and for the interaction effect treatment by group (*F* = 0.861; d*f* = 4290; *p* = 0.488) in the change of the HDRS sum score (see Fig. 1).

**Fig. 2:**
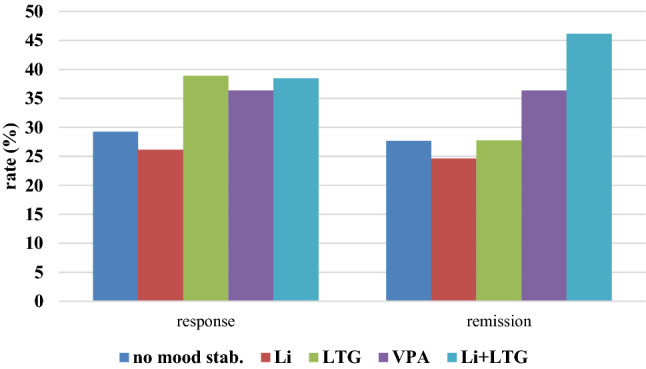
Response and remission rates for patients taking different medication

We also calculated chi-square tests of independence for the variables response/remission and medication group and did not see significant effects (see Fig. 2). Based on direct comparisons between the single groups taking mood stabilizer against the group taking no mood stabilizer, we see a superior effect LTG, VPA and Li + LTG for the response and remission rates only on a descriptive level. This is evident by NNT of 10 for LTG, 14 of VPA and 11 for Li + LTG for response and of 846 for LTG, 12 of VPA and 5 for Li + LTG for remission. Effects were inferior for lithium for response (− 32) and remission (− 33) which is shown by numbers needed to harm (negative NNT).

Grouping the four subgroups taking mood stabilizers together revealed the same findings for the ANOVA (main effect treatment: *p* < 0.001; main effect group: *p* = 0.861; interaction effect: *p* = 0.457). Including relevant covariates also resulted in the same findings (main effect treatment: *p* < 0.001; main effect group: *p* = 0.158; interaction effect: *p* = 0.501).

Relative changes showed no differences between groups. (Cohen’s *d*, [16]), achieved power and needed sample size (equal-sized samples, with an alpha threshold of 5%, two-sided test, power of 80%) using the contrasts groups taking mood stabilizer against the single groups taking no mood stabilizer showed negligible effect sizes and low power for all contrasts (Li: *d* = 0.075, power = 8%, needed sample size of 5566; LTG: *d* = 0.229, power = 15%, needed sample size of 600; VPA: *d* = 0.262, power = 19%, needed sample size of 460) except for the contrast Li + LTG showing medium effect size and power (*d* = 0.581, power = 52%, needed sample size of 96).

## Discussion

Our retrospective analysis suggests that being treated with lithium, LTG or VPA may have no statistically significant or clinically relevant influence on rTMS treatment outcomes in depression. Values for NNT or NNH were low (NNT of 10 for the response rate for LTG means that ten patients would have to be treated with LTG additionally to rTMS to get one more patient with response in contrast to the group with only rTMS). In addition, cell frequencies for LTG, VPA and Li + LTG were low and minimal changes would lead to large changes in NNT or NNH. Thus, effects of this co-medication is at least small or negligible and can only be confirmed in even larger samples sizes.

If validated, this means there is no reason for clinicians to withhold or withdraw these types of medication from patients who are about to undergo a course of rTMS. From own clinical experience, referring physicians and patients are often concerned about these medications attenuating rTMS effects. Our presented data may provide a preliminary evidence base for making that decision in favor of continuing the mood stabilizing agents. This is in line with recent work by Hunter et al. who reported benzodiazepines and psychostimulants to influence rTMS outcomes, but found no effect for antiepileptic drugs [15].

Controlling for confounding factors suggests that age, gender, intake of benzodiazepines or antipsychotics and type of depression do not account for the lack of outcome differences.

**Fig. 3:**
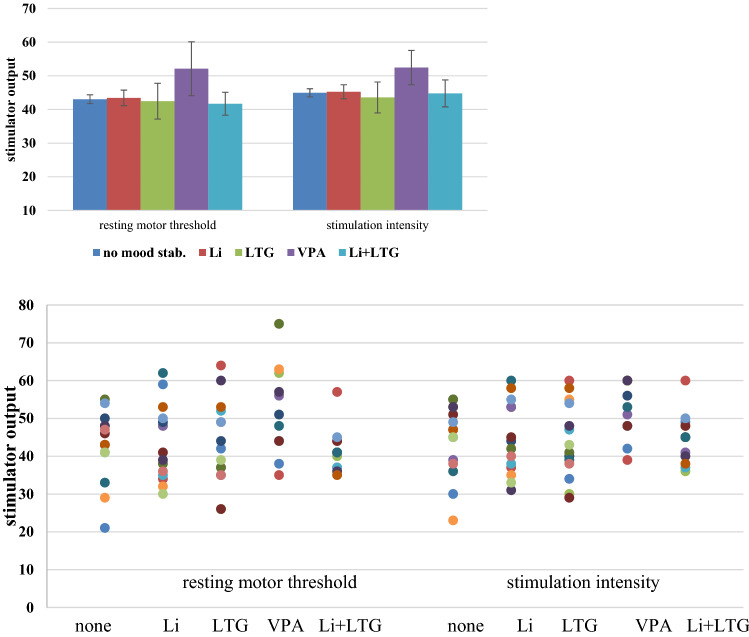
Resting motor threshold and stimulation intensity for patients taking different medication. Error bars represent 95% confidence intervals. **a** Corresponding scatter plot

Motor threshold was significantly and markedly higher for patients taking VPA, fitting the findings in epileptic patients on long-term VPA regimens [2, 18]. This reinforces the hypothesis that long-term administration of the drug has different effects on cortical excitability than single-dosing protocols. This effect was not seen for the other mood stabilizing drugs in our sample. The result is somewhat limited however, since the effect is not sustainable if the outlier (see Fig. 3a) is excluded from analysis, highlighting further the need for larger sample sizes.

Higher MTs did translate into higher stimulation intensities (near significant main effect of group and significant group contrasts) in our patient population on VPA. However, differences between groups were numerically higher for MTs than for stimulation intensities which is due to a ceiling effect, as it is common practice in our rTMS setting to limit treatment intensity to 60% of stimulator output for safety and tolerability reasons. Still, the VPA group did not have worse clinical outcomes despite this intensity limitation. While patient numbers were low for VPA intake, this finding fits with the point made by Minzenberg and Leuchter that antiepileptic drugs such as VPA “may decrease cortical excitability without affecting cortical plasticity” [25].

Strengths of our study include the comparatively large number of patients analyzed and the novelty and potentially large clinical relevancy of the results. The results from our study are compatible with the analysis by Fitzgerald et al. [7], which showed no difference in clinical rTMS outcomes for patients taking versus not taking benzodiazepines, another medication class for which preclinical evidence pointed towards possibly attenuating rTMS effects.

The main limitation of our paper is obviously the methodological issue of retrospective analysis. We still predict prospective controlled work on the matter to be challenging due to ethical and practical considerations. Giving lithium or antiepileptic drugs to depressed patients or withholding them over weeks despite clinical necessity for the purpose of studying rTMS effects provides major ethical and practical challenges.

In case of all drugs except lithium, limited sample size is another issue. Small sample size for the groups taking LTG, VPA and Li + LTG and the unbalanced distribution of the groups stem from the naturalistic prescription practice of these drugs, but of course limit the validity of the results. Achieving adequate statistical power would require even larger clinical samples that ours as stated in the results section. Unequal sample sizes might lead to the missing effect stemming from type II error.

Our practice to limit treatment intensity at 60% stimulator output and the use of comparatively low intensities for medial prefrontal cortex stimulation and the iTBS treatments in this sample might potentially lead to less-than-optimal treatment outcomes when compared to the higher intensities in prospective controlled studies. This in turn may lead to the additional limitation of less sensitivity to detect potential differences between the groups, again owing to the nature of retrospective clinical analysis.

We also do not have systematic data on the grade of treatment resistance of the patients. We do however assume a medium to high level of treatment resistance, as these were inpatients at a tertiary hospital and rTMS is currently practically always used only if several courses of medication have not led to remission.

Since the analysis was performed retrospectively, we had no data on blood levels of the drugs. However, it is common practice in the treatment center to regularly monitor levels of VPA and lithium, so the possibility of noncompliance was at least somewhat mitigated. On the other hand, it is not clear if the effects of long-term VPA on electrophysiological measures are at all dependent on the current blood level or may reflect chronic changes in cortical physiology.

However, despite these limitations the authors are convinced that—given the scarcity of available data on the subject—the analyses presented in this manuscript may provide a rationale for further administration of mood stabilizing drugs when administering rTMS and may serve as a groundwork for further clinical studies.
